# Carotid ultrasound is useful for the cardiovascular risk stratification in patients with hidradenitis suppurativa

**DOI:** 10.1371/journal.pone.0190568

**Published:** 2018-01-04

**Authors:** Marcos A. González-López, Marina Lacalle, Cristina Mata, María López-Escobar, Alfonso Corrales, Raquel López-Mejías, Javier Rueda, M. Carmen González-Vela, Miguel A. González-Gay, Ricardo Blanco, José L. Hernández

**Affiliations:** 1 Division of Dermatology, Hospital Universitario Marqués de Valdecilla, IDIVAL, University of Cantabria, Santander, Cantabria, Spain; 2 Division of Rheumatology, Hospital Comarcal, Laredo, Cantabria, Spain; 3 Division of Rheumatology, Hospital Universitario Marqués de Valdecilla, IDIVAL, University of Cantabria, Santander, Cantabria, Spain; 4 Division of Pathology Hospital Universitario Marqués de Valdecilla, IDIVAL, University of Cantabria, Santander, Cantabria, Spain; 5 Division of Internal Medicine. Hospital Universitario Marqués de Valdecilla, IDIVAL, University of Cantabria, Santander, Cantabria, Spain; University of Palermo, ITALY

## Abstract

**Introduction:**

Hidradenitis suppurativa (HS) is a chronic inflammatory cutaneous disease which has been associated with an increased risk of adverse cardiovascular (CV) outcomes. Adequate stratification of the CV risk is an issue of major importance in patients with HS. To analyze the usefulness of carotid ultrasound (US) assessment for the CV disease risk stratification compared with a traditional score, the Framingham risk score (FRS), in a series of patients with HS.

**Methods:**

Cross-sectional study of 60 patients with HS without history of CV events, diabetes mellitus or chronic kidney disease. Information on CV risk factors was collected and the FRS was calculated. Thus, the patients were classified into low, intermediate and high-CV disease risk categories based on FRS. Carotid US was performed in all participants, and the presence of atherosclerotic plaques was considered as a marker of high CV risk.

**Results:**

HS patients had a mean age of 45.1±10.2 years, and 55% were female. The median FRS was 5.7 (IQR: 3.1–14.7). Twenty-four (40%) of the patients were classified into the low risk group, 28 (46.7%) in the intermediate risk group, and 8 (13.3%) into the FRS-high risk category. Noteworthy, carotid US revealed that about one-third of the patients (17/52; 32.6%) in the FRS-based low and intermediate risk categories had carotid plaques, and, therefore, they were reclassified into a high-risk category.

**Conclusion:**

CV risk in HS patients may be underestimated by using the FRS. Carotid US may be useful to improve the CV risk stratification of patients with HS.

## Introduction

Hidradenitis suppurativa (HS) or acne inversa is a chronic, inflammatory cutaneous disease that affects aproximately 1% to 4% of the general population [1]. It is characterized by the formation of multiples abscesses, nodules and scars in the apocrine gland-bearing areas of the body. The most frequent anatomic sites involved are the axillary, inguinal, perianal, gluteal and submammary regions [1,2]. The etiopathogenesis of HS is not completely understood. Nevertheless, follicular occlusion due to hyperkeratosis is thought to be the primary event, which can lead to subsequent follicular rupture and lymphohistiocytic inflammation [3], with the involvement of the proinflammatory cytokines interleukin (IL)-1 beta, IL-10, Il-12, Il-23 and tumour necrosis factor alpha (TNF-α) [4,5].

HS has been associated with increased prevalence of traditional cardiovascular (CV) risk factors, such as smoking, obesity, dyslipidemia, metabolic syndrome and diabetes mellitus [6–11]. Moreover, an increased risk of adverse CV outcomes has also been reported in HS patients. Thus, a recent population-based cohort study found a significantly higher risk of major adverse CV events (MACEs), including myocardial infarction, ischemic stroke, and CV-associated death in HS patients when compared to controls [12]. In keeping with these findings, we and others have recently demonstrated that patients with HS have an increased prevalence of subclinical atherosclerosis, which persists even after adjusting for classic CV risk factors [13,14]. These observations suggest that HS itself may be an independent risk factor for atherosclerotic CV disease, and support the hypothesis that, as well as in other chronic inflammatory conditions [15–17], the persistent systemic inflammation may be crucial to explain the premature and accelerated atherogenesis in this disorder.

In clinical practice, CV risk assessment has been usually performed by risk scoring systems that incorporate age, sex, diabetes mellitus, serum lipid levels, blood pressure and smoking [18]. These equations are widely used to identify individuals at high risk for developing CV disease events. The Framingham Risk Score (FRS) is a well established coronary risk assesment tool that has been shown to be predictive of CV morbidity and mortality [19]. The Framingham model predicts the risk of developing coronary heart disease (CHD) within 10-year, stratifing patients into three categories (low, intermediate and high-risk) [19,20,21]. There is evidence that risk estimates based on FRS data generalize well to other populations at similar levels of risk, both in the USA and Europe [22]. Although FRS is a widely used method for estimating CV disease risk, it has recognized limitations [23]. Thus, as well as other risk equations, FRS was found to underestimate the actual CV risk in patients with several chronic inflammatory diseases, such as rheumatoid arthritis [24], ankylosing spondilitis [25,26] and psoriasis [27,28].

Several imaging techniques are currently available to disclose the presence of atherosclerotic macrovascular disease. Carotid ultrasonography (US) is a validated tool for the evaluation of subclinical atherosclerosis through the assessment of cIMT and atheroma plaques [29–31]. The finding of carotid atherosclerotic plaques was found to be a good predictor of CV events in low and intermediate risk groups of individuals with non-rheumatic disorders [32], and their presence automatically put the patient on a very high CV risk category [33].

Taking together all these considerations, in the present study we have compared the CV risk estimated by a traditional score, the FRS, with that obtained by adding carotid US assessment of subclinical atherosclerosis in patients with HS. Thus, we aimed to determine if the use of carotid US may improve the stratification of the CV risk of HS patients.

## Patients and methods

### Patients

In this cross-sectional study, 60 patients with a diagnosis of HS were recruited at the University Hospital Marqués de Valdecilla in Santander, Spain. The diagnosis of HS was done by dermatologists in all patients.

For the purpose of this study, we used the methodology that we previously described in detail [13]. Thus, patients with a history of CV events (CHD, cerebrovascular disease, heart failure, and/or peripheral arterial disease) were excluded. Furthermore, patients with type 1 and type 2 diabetes mellitus were also excluded, since diabetes is considered as a CHD risk equivalent. Furthermore, we did not include patients with chronic kidney disease (defined as a glomerular filtration rate <60 ml/min/1.73 m^2^), since these are also considered a group with high CV risk. We also excluded HS patients who had another concomitant inflammatory disease, such as inflammatory bowel disease, inflammatory arthritis (rheumatoid arthritis or spondyloartropaties), connective tissue diseases (systemic lupus erythematosus or other autoimmune diseases) or cutaneous inflammatory diseases (psoriasis or atopic dermatitis among others).

A clinical examination was carried out in all patients. The severity of HS was assessed by the HS Physician's Global Assessment (HS-PGA), which includes 6 stages (scale 0–5) with clear guidance for disease severity assessment [13,34]. According to HS-PGA, HS was classified as moderate-severe-very severe when PGA≥3. Moreover, information on disease duration was also assessed in all patients.

The study was approved by the Ethical Committee of Cantabria (Spain). Written consent was obtained from all participants.

### Cardiovascular risk factors assessment

All patients provided information on their demographics, past medical history, smoking status, family history of early CHD in first-degree relatives and use of antihypertensive medications and/or lipid-lowering agents. Traditional CV risk factors were defined as previously reported [13]. Body height and weight, body mass index (BMI), systolic blood pressure (BP) and diastolic BP were measured in all patients at the time of the study. Blood samples were drawn after an overnight fast, and serum total cholesterol (TC), high-density lipoprotein cholesterol (HDL-c), low-density lipoprotein cholesterol (LDL-c), triglycerides, glucose, high-sensitivity C-reactive protein (hs-CRP) levels, and erythrocyte sedimentation rate (ESR) were assessed.

### Framingham risk scoring

We used the ATP III charts [19] to calculate the 10-year CHD risk for each patient. Risk factors considered were: age at the time of the study, gender, smoking, systolic BP, use of antihypertensive treatment, and TC and HDL-c concentrations. As proposed by Greenland et al, [35] each patient was classified into one of the following three risk categories: low risk (≤5%), intermediate risk (6–20%) and high risk (>20%).

### Carotid ultrasound examination

As described in detail previously [13], the detection of carotid atherosclerotic plaques in the extracranial tree was performed using a commercially available scanner, Mylab 70 Esaote (Genoa, Italy) equipped with a 7 to 12 MHz linear transducer and the automated software-guided technique radiofrequency-Quality Intima Media Thickness in real-time (QIMT, Esaote, Maastricht, The Netherlands). Carotid atherosclerotic plaque was identified as recommended in the Mannheim consensus, that is when a focal structure that encroaches into the arterial lumen of at least 0.5 mm or 50% of the surrounding cIMT value or demonstrates a thickness of >1.5 mm as measured from the media-adventitia interface of the intima-lumen interface, is present [36]. The presence of carotid plaques was considered as the gold standard of severe subclinical atherosclerosis.

### Statistical analysis

Results were reported as mean ± standard deviation (SD), median and interquartile range (IQR) or proportions as appropriate. The association of quantitative recorded variables with carotid plaque was assessed by means of Student t-test or Mann-Whitney U-test, as appropriate. Pearson chi-square test or Fisher exact test were used to compare qualitative variables.

The performance of the FRS in assessing plaque presence was determined by the area under the curve (AUC) in ROC curve analysis. To determine clinically useful cutoff values in assessing the presence of plaque, we set the sensitivity and specificity of FRS equation at 80% in sequential analyses. We also determined the sensitivity and specificity when the conventional cutoff values of 5% and 20% were used for the FRS in assessing plaque presence. Two-sided p<0.05 was considered statistically significant. STATA 12/SE software (StataCorp, College Station, TX) was used in all the calculations.

## Results

### Baseline characteristics

Sixty consecutive Caucasian patients with HS were studied. The baseline characteristics of the study population according to the presence of plaque are listed in the Table 1. The mean age of the patients at the time of the study was 45.1±10.2 years, and 55% were women. The median (IQR) disease duration was 18 (10–27) years. Thirty-six patients (60%) were classified as moderate-severe/very severe HS (HS-PGA≥3), and the remainder 24 (40%) as minimal-mild HS (HS-PGA <3). Twenty-three (38.3%) patients were currently being treated with TNF inhibitors.

**Table 1:** Baseline features of HS patients according to the presence of carotid plaque.

	Total(n = 60)	Without Plaque(n = 38)	With Plaque(n = 22)	p
**Demographics**				
*Age*, *years*	45.1±10.2	42.9±10.5	48.8±8.8	**0.03**
*Women*	33 (55.0)	20 (52.6)	13 (59.1)	0.63
*Weight*, *Kg*	81.9±17.1	83.7±17.6	78.9±16.1	0.31
**Conventional CV risk factors**				
*Hypertension*	11 (18.3)	6 (15.8)	5 (22.7)	0.51
*Dyslipidemia*	9 (15.0)	5 (13.5)	4 (18.2)	0.72
*Systolic blood pressure*, *mm Hg*	132.7±16.7	131.5±16.5	134.6±17.4	0.50
*Diastolic blood pressure*, *mm Hg*	80.7±9.7	80.9±9.9	80.5±9.5	0.89
*Total cholesterol*, *mg/dl*	188.8±31.7	187.2±31.0	191.5±33.4	0.62
*LDL-cholesterol*, *mg/dl*	117.8±32.3	115.9±31.1	121.1±34.8	0.55
*HDL-cholesterol*, *mg/dl*	51.1±16.7	51.2±16.4	50.8±17.6	0.93
*Cholesterol/HDL-cholesterol ratio*	3.97±1.21	3.9±1.2	4.1±1.3	0.65
*Triglycerides*, *mg/dl*	97.7±44.8	97.7±49.0	97.7±37.6	0.99
*Current smoking*	37 (61.7)	19 (50.0)	18 (81.8)	**0.02**
*Body mass index > 30 Kg/m*^*2*^	25 (41.7)	18 (47.4)	7 (31.9)	0.37
*Family history of early CHD in first-degree relatives*	7 (11.7)	5 (13.2)	2 (9.1)	0.96
**CV drug use**				
*Antihypertensive drugs*	4 (6.7)	2 (5.3)	2 (9.1)	0.62
*Lipid-lowering agents*	7 (11.6)	4 (10.5)	3 (13.6)	0.96
**Risk factor control**				
*Blood pressure < 140/90 mmHg*	41 (68.3)	26 (68.4)	15 (68.2)	0.98
*LDL-cholesterol < 70 mg/dl*	3 (5.0)	2 (5.3)	1 (4.5)	0.99
**HS features**				
*Duration*, *yrs*	18.0 (10.0–26.8)	15.5 (7.0–25.0)	23.5 (15.3–30.3)	**0.02**
*PGA (≥3)*	36 (60.0)	19 (50.0)	17 (77.3)	**0.04**
*CRP*, *mg/l*	0.35 (0.15–0.74)	0.35 (0.16–0.72)	0.37 (0.15–0.89)	0.81
*ESR*, *mm/1*^*st*^ *h*	13.5 (6.0–22.0)	9.0 (3.0–22.5)	15 (7.8–22.5)	0.27
**cIMT, mm**	0.622±0.099	0.603±0.093	0.656±0.104	**0.04**
**Framingham score**	5.7 (3.1–14.7)	4.8 (2.8–11.3)	7.9 (4.9–19.9)	**0.04**

PGA, physician global assessment; ESR, erythrocyte sedimentation rate; CRP, C-reactive protein; SD, standard deviation; cIMT, carotid intima-media thickness; CV, cardiovascular.Values are expressed as mean±SD, median (interquartile range) or percentages and compared by means of Chi2-test, Mann-Whitney U-test, Student t-test or Fisher test as appropriate. Significant values are marked in bold.

### Framingham risk score

The mean (IQR) FRS of the study population was 5.7 (3.1–14.7). There were 24 (40%) HS patients who were classified into the FRS-low risk group, 28 (46.7%) into the intermediate risk group, and 8 (13.3%) into the high risk category.

### Carotid ultrasound findings

Carotid plaques were present in 22 HS patients (36.6%). As shown in the Table 1, those with plaques were older (48.8±8.8 versus 42.9±10.5 years; p = 0.03) and had a significantly longer disease duration (23.5 versus 15.5 years; p = 0.02) than those without. They had more commonly severe forms of HS (HS-PGA ≥3), and were more frequently current smokers than HS patients without carotid plaques. Also, cIMT values were significantly higher in patients with plaques than those without (0.656±0.104 mm versus 0.603±0.093 mm; p = 0.04). The mean FRS for HS patients with carotid plaques was 7.9 (4.9–19.9), which was significantly higher than the mean value for those without plaques [4.8 (2.8–11.3); p = 0.04].

### Comparison between the FRS and carotid US assessment in the CV risk stratification

**Fig 1:**
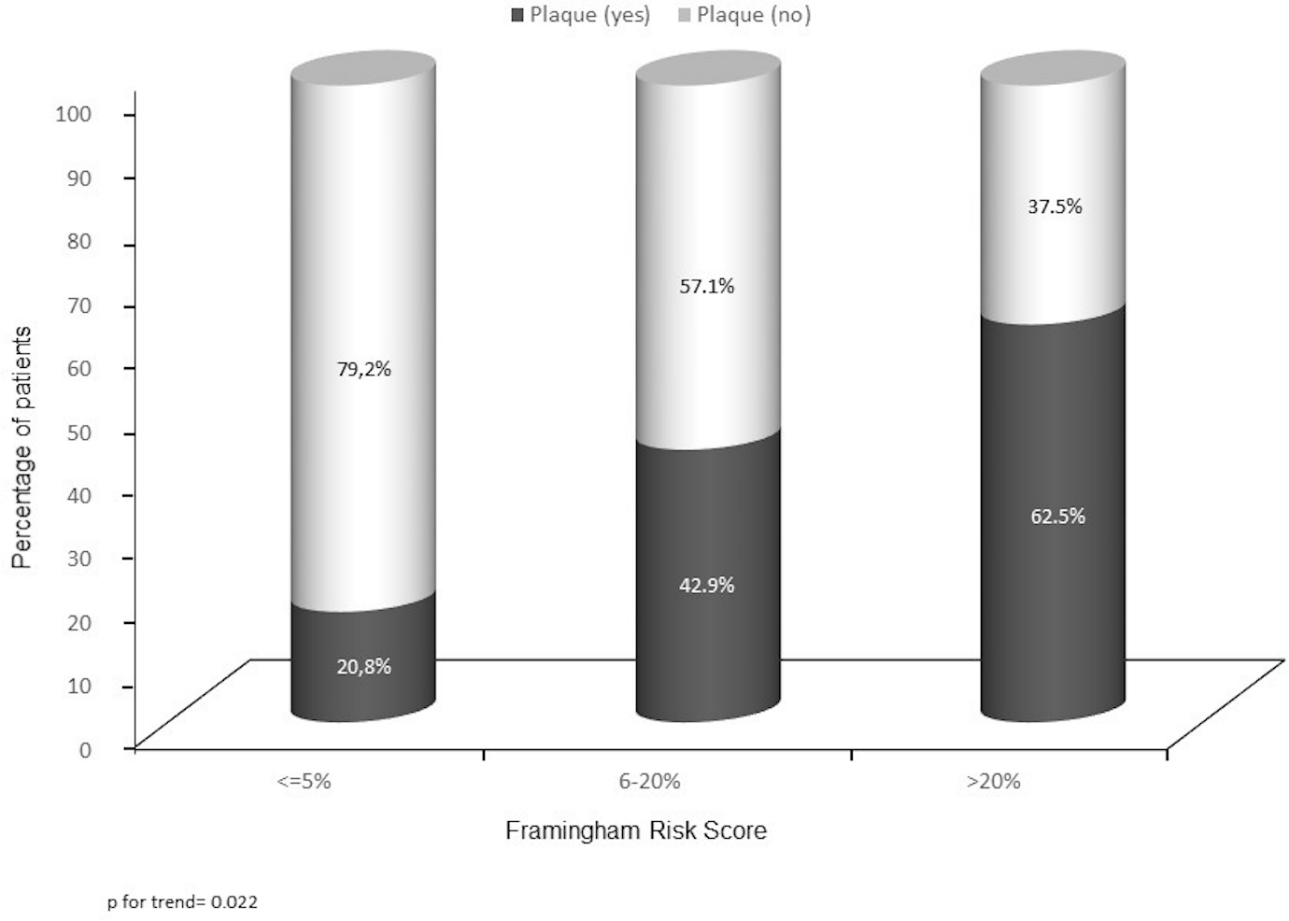
Frequency of carotid plaques by each Framingham risk score based category.

The frequency of carotid plaques by each FRS-based category was calculated to estimate the ability of the Framingham equation to correctly classify patients as having high CV risk. Five (20.8%) of the HS patients who fulfilled the low CV risk category had carotid plaques, and were reclassified into an ultrasound-based high-CV disease risk group. Moreover, 12 (42.9%) of the patients from the FRS-based intermediate-risk category had also plaques, and so, they were reclassified into an ultrasound-based high- CV disease risk group (Fig 1). Therefore, carotid US correctly reclassified as high risk patients, about a third (17/52; 32.6%) of the patients included in the intermediate or low risk categories according to FRS.

### ROC curve analysis on the CVD risk equation-carotid plaque relations

**Fig 2:**
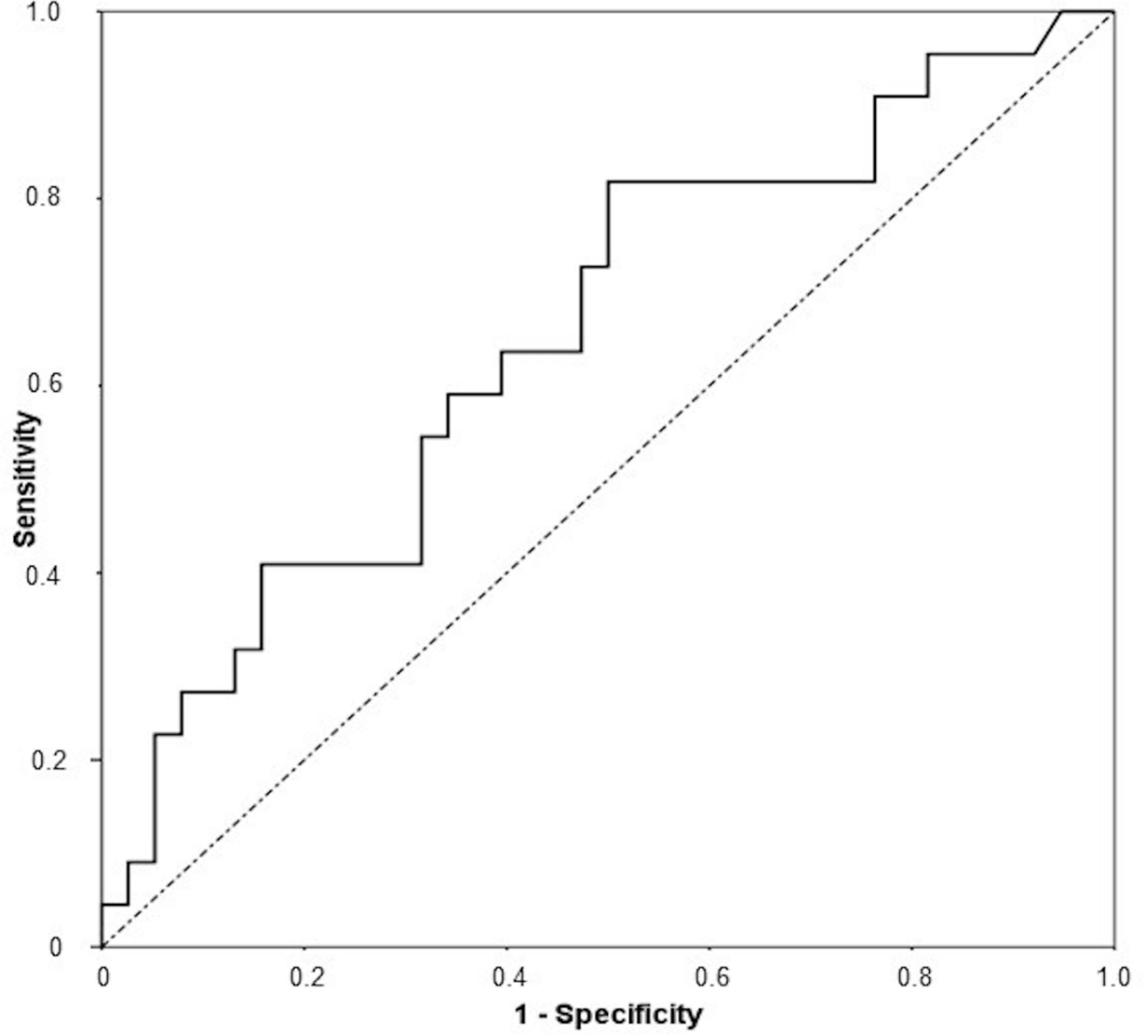
ROC curve for the relationship between Framingham risk score and carotid plaque.

ROC curve for the relationship between FRS and carotid plaque measured by US is shown in the Fig 2. The AUC (95% CI) was 0.65 (0.51–0.80); p = 0.04. Table 2 shows that when sensitivity for the FRS was set at 80%, the cut-off value was 4.8%, with a corresponding specificity, positive predictive value (PPV), negative predictive value (NPV), and correct classification percentage of 50%, 49%, 83% and 61%, respectively. When specificity was set at 80%, the cut-off value was 13.3% and the corresponding sensitivity, PPV, NPV, and correct classification percentage were 40%, 80%, 53%, and 65%, respectively. At a conventional high risk cut-off of 20% for FRS, the corresponding sensitivity, specificity, PPV, NPV and correct classification percentage were 23%, 92%, 63%, 67% and 67%, respectively.

**Table 2:** Classification of Spanish patients with HS at Framingham risk equation cutoff values based on sensitivity or specificity set at 80% in ROC analysis, or conventional recommendations.

	Cutoff value (%)	Sensitivity (%)	Specificity (%)	PPV(%)	NPV(%)	Correct classification (%)
Framingham score	≥ 4.8	80 (63–100)	50 (33–67)	49 (31–66)	83 (65–100)	61
	≥ 13.3	40 (18–63)	80 (65–93)	53 (26–79)	70 (55–85)	65
	≥ 20	23 (3–42)	92 (82–100)	63 (23–100)	67 (54–81)	67

PPV, Predictive positive value; NPV, Negative predictive value. CI 95% in parentheses.

## Discussion

The results observed in the current study indicate that a traditional CV risk algorithm, the FRS, has limitations in its ability to correctly stratify subjects into CV disease risk groups and underestimates the actual risk in patients with HS. A substantial number of HS patients who were classified in the low and intermediate risk groups according to the FRS had atherosclerotic plaques, and therefore, they were reclassified into a high-risk category, after carotid US assessment was performed.

Atheromatous plaque formation indicates intimal pathological changes, and is considered a later step in the atherogenesis process more closely linked to CHD risk factors and myocardial infarction compared to increased cIMT [37]. Moreover, the detection of atherosclerotic plaques increased the predicted CHD risk at any level of cIMT [30], and subjects with carotid plaques should be automatically considered as very high CV disease risk individuals [26,33].

There are several possible reasons that could explain the discordance between the FRS and carotid US findings. Firstly, CV scoring systems are aimed to predict the risk for CHD or total CVD risk, but a significant percentage of patients with severe atherosclerosis will be missed when those scoring systems are used as the initial risk assessment [38]. With respect to this, the FRS predicts CV risk morbidity and mortality but it has not been tested for the prediction of subclinical atherosclerosis. In this regard, the FRS uses only standard risk factors, but other factors such as obesity, insulin resistance and metabolic syndrome also can contribute to the development of atherosclerosis and are not captured in the scoring system [23,39].

Secondly, the problem of CV disease risk underestimation with these risk calculators is increased in patients with chronic inflammatory diseases. In these patients, chronic systemic inflammation may promote the development of endothelial cell dysfunction with the subsequent development of accelerated atherogenesis [13]. In this sense, several studies have shown that the FRS and other CV risk algorithms often underestimate the CV risk in patients with rheumatic diseases, such as rheumatoid arthritis and ankylosing spondylitis [24–26], when tested against imaging findings of subclinical atherosclerosis. Moreover, a recent study revealed that 55.9% of psoriatic patients with intermediate CV risk according to the FRS had carotid plaques [27]. Our results in patients with HS confirm and extend these findings observed in patients with other chronic inflammatory conditions.

Our study shows that carotid US may be useful as an additional tool to improve the sensitivity of the FRS to detect HS patients at high CV disease risk. Because of that, we feel that the use of non-invasive imaging techniques to disclose the presence of subclinical atherosclerosis may complement currently used risk assessment tools. An adequate stratification of HS patients would provide a more tight CV disease risk factor control in a clinically relevant proportion of these patients that are included in the categories of low and intermediate estimated CV disease risk according to FRS. It is of particular relevance if we consider that patients at high CV risk as evidenced by carotid US-determined plaque presence require intensive CV disease risk management. In this regard, the European Guidelines on cardiovascular disease prevention in clinical practice recommend intensive lipid lowering treatment with a LDL cholesterol target of <1.8 mmol/L (<70 mg/dl) in individuals with very high CV disease risk [33]. In this regard, our data also highlight that low FRS cut-off values, close to 5%, have a high sensitivity to detect high-risk subclinical atherosclerosis in patients with HS. In fact, with a FRS cut-off value of 20%, sensitivity was further reduced to 23%.

The limitations of our study include an overall small size sample and those inherent to a cross-sectional design. Prospective longitudinal studies are needed to determine whether reclassification to a higher category actually translates to increased CV events in patients with HS.

In summary, the results of the current study suggest that carotid US may improve CV disease risk stratification of patients with HS. The use of this non-invasive technique allows us to identify HS individuals at high risk who would benefit of having a strict management of CV risk factors. Based on our data, we recommended the use of carotid US in HS-patients with an intermediate risk for cardiovascular disease (40% of prevalence of carotid plaques in our series). Low-risk HS individuals aged 50 years and older, active smokers, with severe (PGA ≥3) or longstanding disease (>20 years) should be considered for carotid ultrasonography assessment according to an individualized clinical evaluation.

## Supporting information

S1 File DATA POINTS-PONE-D-16-42830R1. (XLSX)
